# Anti-Aflatoxigenic *Burkholderia contaminans* BC11-1 Exhibits Mycotoxin Detoxification, Phosphate Solubilization, and Cytokinin Production

**DOI:** 10.3390/microorganisms12091754

**Published:** 2024-08-23

**Authors:** Lixia Hua, Pengsheng Ye, Xue Li, Hanhong Xu, Fei Lin

**Affiliations:** 1 Industrial Crop Research Institute, Sichuan Academy of Agricultural Sciences, Chengdu 610300, China; 2 Key Laboratory of Integrated Pest Management on Crops in Southwest of Ministry of Agriculture and Rural Affairs, Chengdu 610066, China; 3 State Key Laboratory for Conservation and Utilization of Subtropical Agro-Bioresources, South China Agricultural University, Guangzhou 510642, China; 4 Key Laboratory of Natural Pesticide and Chemical Biology, Ministry of Education, South China Agricultural University, Guangzhou 510642, China

**Keywords:** biocontrol agent, mycotoxin detoxification, biofertilizer, phosphate-solubilizing microorganisms, phytohormone

## Abstract

The productivity and quality of agricultural crops worldwide are adversely affected by disease outbreaks and inadequate nutrient availability. Of particular concern is the potential increase in mycotoxin prevalence due to crop diseases, which poses a threat to food security. Microorganisms with multiple functions have been favored in sustainable agriculture to address such challenges. *Aspergillus flavus* is a prevalent aflatoxin B_1_ (AFB_1_)-producing fungus in China. Therefore, we wanted to obtain an anti-aflatoxigenic bacterium with potent mycotoxin detoxification ability and other beneficial properties. In the present study, we have isolated an anti-aflatoxigenic strain, BC11-1, of *Burkholderia contaminans*, from a forest rhizosphere soil sample obtained in Luzhou, Sichuan Province, China. We found that it possesses several beneficial properties, as follows: (1) a broad spectrum of antifungal activity but compatibility with *Trichoderma* species, which are themselves used as biocontrol agents, making it possible to use in a biocontrol mixture or individually with other biocontrol agents in an integrated management approach; (2) an exhibited mycotoxin detoxification capacity with a degradation ratio of 90% for aflatoxin B_1_ and 78% for zearalenone, suggesting its potential for remedial application; and (3) a high ability to solubilize phosphorus and produce cytokinin production, highlighting its potential as a biofertilizer. Overall, the diverse properties of BC11-1 render it a beneficial bacterium with excellent potential for use in plant disease protection and mycotoxin prevention and as a biofertilizer. Lastly, a pan-genomic analysis suggests that BC11-1 may possess other undiscovered biological properties, prompting further exploration of the properties of this unique strain of *B. contaminans*. These findings highlight the potential of using the anti-aflatoxigenic strain BC11-1 to enhance disease protection and improve soil fertility, thus contributing to food security. Given its multiple beneficial properties, BC11-1 represents a valuable microbial resource as a biocontrol agent and biofertilizer.

## 1. Introduction

Crops are constantly exposed to different biotic and/or abiotic stresses that strongly impact crop yield and quality. Phytopathogen infection and nutrient deficiency are key factors affecting global agricultural productivity and food security. Disease-related production losses are estimated to range from 21.5% to 50% worldwide, thus having a major economic impact [1]. Crop diseases also pose a considerable threat to human and animal health, as some filamentous fungi produce mycotoxins, including aflatoxins (AFs), zearalenone (ZEN), ochratoxins, deoxynivalenol, and patulin, once they colonize and proliferate in crops [2,3,4]. There are significant regional and global variations in the occurrence of mycotoxins in food crops with an overall prevalence ranging from 60 to 80% [5]. Notably, AFs produced by the *Aspergillus* genus are the most toxic and carcinogenic [6].

*Aspergillus flavus* is one of the main aflatoxin-producing fungi present in a wide range of crops primarily grown in warm and humid climates. Strategies such as resistance breeding, pesticide application, and biological control have been used to mitigate *A. flavus* infections and AF contamination [7]. However, prolonged reliance on synthetic, chemical fungicides has had an adverse impact on the environment and human health, prompting an increased emphasis on biocontrol as an alternative, sustainable control strategy [8,9,10]. Non-aflatoxigenic *A. flavus* (AF^−^) strains have been utilized for many years as effective biocontrol agents to reduce AF contamination in crops [11]. Nonetheless, their ecological safety, genetic stability, and limitations in performance must be considered, necessitating the identification of additional biological control agents [12]. Therefore, investigating anti-aflatoxigenic microorganisms and their natural products for significant anti-aflatoxigenic activity is crucial. While numerous beneficial microorganisms have been studied in recent years, only a few exhibits significant inhibitory activity against *Aspergillus* and also possess both antifungal and mycotoxin detoxification properties [13]. Thus, the multifunctional properties of beneficial microorganisms should be emphasized, including their antifungal spectrum and ability to detoxify mycotoxins, the spectrum of their detoxification properties, and other beneficial characteristics.

Phosphorus (P) is one of the main growth-limiting macronutrients required by crop plants for maximum yields. However, crops, on average, only utilize 20–25% of the total amount of phosphate fertilizers applied, and this can be as low as 10% in some intensively managed crops [14,15]. Inorganic phosphorus predominates in soil, yet limited phosphorus uptake remains a significant global constraint on agricultural productivity and food security [16]. Improving crop P consumption is essential for conserving P resources [17]. In this regard, phosphate-solubilizing microorganisms are crucial to the natural P cycle, in which they transform fixed phosphate in the soil into phosphate forms that plants can use. Thus, they are seen as being foundational to sustainable agriculture [15,18]. Microbes can solubilize a portion of the insoluble P, making it available to plants, in several diverse ways [19,20,21,22]. Phosphate-solubilizing bacteria play a crucial role in accumulating and transforming phosphorus, thereby enhancing its uptake for plant growth. They offer a cost-effective and sustainable biological approach to addressing soil phosphorus deficiency [23,24].

Plant growth-promoting bacteria (PGPB) are beneficial bacteria that inhabit the rhizosphere, rhizoplane, and roots of plants [25]. PGPB enhance plant growth through direct and/or indirect mechanisms, including phosphate solubilization, phytohormone production, and the inhibition of phytopathogens [26,27]. As alternative fertilizers, the use of PGPB has emerged as a principal method of supporting sustainable agricultural development by reducing the need for synthetic pesticides and fertilizers while improving soil quality [28]. A variety of PGPB exhibit diverse functional mechanisms and impact different crops differently [29]. Research has demonstrated that indigenous strains of PGPB are more likely to thrive in the soil environment when they are introduced into the plant rhizosphere, which increases their tolerance to local environmental stresses, a feature that may be critical given present projected climate change scenarios [30,31].

The objective of the present study was to isolate indigenous strains of anti-aflatoxigenic PGPB and evaluate their ability to support sustainable agriculture development. This included an assessment of properties such as their antifungal spectrum, ability to detoxify mycotoxins, ability to make nutrients more readily available to plants, and capacity to synthesize phytohormones. The discovery of multifunctional strains of beneficial bacteria holds great potential for the development of new types of biofertilizers.

## 2. Materials and Methods

### 2.1. Anti-Aflatoxigenic Strain Isolation

Ten soil samples were collected in June 2021 in Sichuan Province from the rhizosphere of plants located on farms and woods. Bacteria were isolated from soil samples by adding 1 g of soil to 10 mL of sterile water, followed by shaking the mixture at 180 rpm for 1 h. Subsequently, 500 μL of suspension was transferred into sterile water to obtain a 10^−4^ dilution. A total of 100 μL of the diluted sample was plated on a Luria–Bertan (LB) agar plate (10 g/L tryptone, 5 g/L yeast extract, 10 g/L NaCl, and 15 g/L agar) and incubated at 28 °C for 3 d. Single colonies were inoculated into 1.5 mL of LB liquid medium and incubated overnight at 28 °C and 130 rpm. The inhibition zone method was used to assess anti-aflatoxigenic activity. A total of 20 µL of a conidial suspension (10^5^ cfu/mL) of *Aspergillus flavus* isolated from peanut kernels was spread over a potato dextrose agar (PDA) plate. Subsequently, 50 µL of a bacterial suspension was administered into a hole in the middle of the PDA plates, which were then incubated overnight at 28 °C. The inhibition activity was measured based on the clear zone diameter.

### 2.2. Anti-Aflatoxigenic Activity of the Cell-Free Supernatant

Cell-free culture supernatant (CFS) was obtained from an anti-aflatoxigenic strain (BC11-1) cultured for 24 h to assess anti-aflatoxigenic activity. The CFS was obtained by filtering the BC11-1 suspension through a 0.22 μm sterile filter. A total of 50 mL of sterile PDA was combined with 25 mL of cell-free culture filtrate and then poured into Petri plates. Next, a 0.5 cm-diameter PDA plug of *A. flavus* mycelia was placed in the middle of the PDA plates, and the plates were incubated at 28 °C for 7 d. The inhibition ratio (%) was calculated using the following formula: growth inhibition (%) = [(colony radius of the pathogen in control plate − colony radius of the pathogen in inhibition plate)/colony radius of the pathogen in control plate] × 100.

*A. flavus* hyphae were cultured in YG broth (glucose 30 g/L and yeast extract 5 g/L) with shaking at 130 rpm for 2 d at 28 °C. Mycelia were collected by pouring the culture through filter paper. The mycelia were then washed twice with phosphate-buffered solution (PBS) and treated with 1 mL of CFS at 28 °C for 48 h. Samples treated with CFS were then processed for transmission electron microscopy (TEM) (JEOL, Tokyo, Japan).

### 2.3. Antifungal Spectrum of BC11-1

Nine fungi, including seven phytopathogens and two *Trichoderma* biocontrol agents, were selected for testing the inhibitory activity of BC11-1. The seven phytopathogens included *Botrytis cinerea* isolated from eggplant, *Fusarium solani* isolated from peony, *Phomopsis asparagi* isolated from asparagus, *Fusarium graminearum* isolated from wheat, and *Rhizoctonia solani*, *Alternaria tenuissima*, and *Curvularia oryzae* isolated from rice. The biocontrol agents, *Trichoderma virens* strain T23 and *Trichoderma harzianum* strain T22, were isolated from a soil sample from China [32] and a granule commercial formulation [33], respectively. *Trichoderma* strains were used to assess the compatibility of BC11-1 with other biocontrol agents. The different fungi were inoculated on PDA plates and cultured at 21–28 °C until they covered the entire Petri dish. Subsequently, four mycelial plugs of 0.5 cm in diameter were positioned on new PDA plates in a cross shape. A single colony of the antifungal bacterium was inoculated into 10 mL of LB liquid medium and shaken at 28 °C, 130 rpm overnight until the absorbance (OD, optical density value) at a 600 nm wavelength reached 1.0 (OD_600_ = 1.0). Subsequently, 50 µL of suspension was inoculated into holes in the middle of the PDA plates containing various types of fungi. The inhibition ratio was determined with the formula mentioned above. The test was repeated four times.

### 2.4. Determination of the Mycotoxin Detoxication Capacity of BC11-1

A 0.22 μm sterile filter membrane was placed on the surface of an LB plate containing 1 μg/mL AFB_1_ or ZEN mycotoxins to determine the detoxication capacity of BC11-1. A total of 100 μL of a BC11-1 suspension cultured overnight (OD_600_ = 0.8) was evenly coated on the filter membrane to separate bacterial cells from the mycotoxins. After co-culturing at 28 °C for 7 d, the sterile filter membrane containing BC11-1 cells on the plate was discarded, and mycotoxin content in the LB plate was determined using a commercially available enzyme-linked immunosorbent test kit (Sangon Biotech, Shanghai, China).

A piece of filter paper was placed on a plate. Subsequently, 15 g of corn seeds were placed on the filter paper after disinfection with 75% ethyl alcohol. Two plates were prepared for this assay. Both plates were inoculated with one loop of *A. favus* spores. In one plate, a corn seed was covered with 4 mL of CFS obtained from a BC11-1 culture collected after 48 h of fermentation. Corn seeds treated with an equal volume of sterile water served as a control. Plates were sealed with sealing film and cultured at 28 °C for 7 d to assess the inhibitory activity of BC11-1 against *A. favus*. Aflatoxin production in each plate was determined with a commercially available enzyme-linked immunosorbent test kit (Sangon Biotech, Shanghai, China).

### 2.5. Biochemical Characterization and Extracellular Substance Identification

The anti-aflatoxigenic BC11-1 strain was cultured overnight in 20 mL of LB liquid medium at 28 °C with shaking at 130 rpm, followed by restreaking on LB agar and nutrient agar plates to obtain single colonies. A Biolog GENIII MicroPlate (Biolog, Inc., Hayward, CA, USA) was inoculated with a suspension of BC11-1 and incubated at 33 °C with shaking at 130 rpm to obtain an absorbance of 90% and then analyzed. The test was repeated twice.

The activity of the extracellular substances of BC11-1 was assessed using the hole method, involving the inoculation of a 50 µL overnight cultured suspension into a 0.5 cm-diameter hole in the center of agar plates and incubating them at 28 °C. The presence of a distinct halo around the hole was considered indicative of positive activity. The National Botanical Research Institute’s phosphate growth medium (NBRIP) comprising 10 g/L D-glucose, 0.5 g/L (NH_4_)_2_SO_4_, 0.3 g/L NaCl, 0.3 g/L MgSO_4_·7H_2_O, 5 g/L Ca_3_(PO_4_)_2_, 0.03 g/L FeSO_4_·7H_2_O, 0.03 g/L MnSO_4_·H_2_O, and 0.3 g/L KCl was used to determine phosphate solubilization activity [34]. Extracellular protease activity was measured on skim milk agar plates [35]. Siderophore production was determined using the methodology of Schwyn and Neilands [36]. Amylase activity analysis was assessed on soluble starch agar plates supplemented with 10.0 g/L tryptone, 5.0 g/L NaCl, and 2.0 g/L soluble starch. Results were visualized using Lugol’s iodine solution (0.5% I_2_ and 1% KI, *w*/*v*). β-1 and 3-glucanase activity characterization was performed on agar plates containing 1.0 g/L D-glucose, 1.0 g/L K_2_HPO_4_, 3.0 g/L Na_2_HPO_4_, 0.5 g/L MgSO_4_·7H_2_O, 4.0 g/L Poria cocos powder, and 0.06 g/L aniline blue.

### 2.6. Phosphate Solubilization Efficiency

A solitary colony of the BC11-1 strain was introduced into 10 mL of LB liquid medium and cultured overnight at 28 °C on a rotary shaker (Eppendorf, Hamburg, Germany) set at 130 rpm. Subsequently, 250 mL of NBRIP liquid medium, augmented with 1 mL of the culture suspension, was added, and the mixture was shaken at 135 rpm for 0, 1, 2, 3, 4, 7, and 10 d at 28 °C. After centrifugation at 8000 rpm for 5 min, 30 mL of fermentation broth was filtered through a 0.22 µm Millipore filter (Millipore, Boston, MA, USA) to eliminate bacteria and insoluble calcium phosphate. The concentration of soluble phosphorus in the filtrate was then determined using inductively coupled plasma-optical emission spectrometry (ICP-OES, ICPE-9820, Shimadzu, Kyoto, Japan). The pH of each culture filtrate was also assessed. A statistical analysis was performed using a single-factor analysis of variance with Tukey’s test in Data Processing System software (Version 9.01, Zhejiang University, Hangzhou, China) [37]. The analysis was conducted four times.

### 2.7. Greenhouse Experiment and Total Phosphorus Determination

Surface-disinfected rice seeds (a hybrid rice variety Taiyou 808, immersion in 75% ethyl alcohol for 30 s) were placed in Petri plates to initiate germination. The germinated seeds were then separated into the following three groups for planting: Group I received tap water only and served as a negative control; Group II received tap water containing 5 g/L Ca_3_(PO_4_)_2_; and Group III received water containing either 60 mL or 180 mL phosphate solution produced by BC11-1 strain 4 d earlier, as previously described. All liquids were subjected to a 1 h incubation at 50 °C prior to use to deactivate bacterial activity. The seedlings were then transplanted into a greenhouse for water culture at 28 °C and a 13:11 light/dark photoperiod. After 14 d, the whole plants, including the leaves and roots, were harvested and dried. Subsequently, 0.2 g of dry sample from each group was digested in 10 mL of 68% HNO_3_ in a microwave digestion instrument (Touchwin2.0, APL Technology Co., Ltd., Chengdu, China) utilizing the following sequential protocol: first step at 120 °C for 5 min, second step at 140 °C for 5 min, third step at 160 °C for 20 min, fourth step at 170 °C for 20 min, and fifth step at 180 °C for 20 min.

All digests were placed in an acid-driven processor (Sineo Co., Ltd., Shanghai, China) with 1000 W of applied power. The acid was evaporated at 90 °C until only 1–2 mL of digest liquid remained. All digests were then diluted to a volume of 25 mL with 1% HNO_3_ for use in the previously described ICP-OES assay to determine phosphorus content. The test was repeated three times.

### 2.8. Cytokinin Production by BC11-1

A single colony of BC11-1 was introduced into 100 mL of LB liquid medium and cultured at 28 °C on a rotary shaker set at 120 rpm for 48 h, 72 h, 96 h, and 120 h. Subsequently, 5 mL of cell-free culture filtrate and 1 g of bacterial cells from each treatment were analyzed for cytokinin using high-performance liquid chromatography-mass spectrometry (LC-MC) with an Agilent 1290 HPLC system (AB Sciex, St. Louis, MO, USA). Separation was achieved using a Poroshell 120 SB C18 column (50 × 2.1 mm, 2.7 μm), with an injection volume of 2 μL. The mobile phase comprised methyl alcohol (A) and water containing 0.1% formic acid (B) at a flow rate of 0.3 mL/min. Mass spectrometry assessments were conducted utilizing a Qtrap6500 mass spectrometer system in positive and negative ion modes, with instrument parameters set as follows: gas temperature 400 °C and ion spray voltage +4500 V, −4000 V. Mass spectra were acquired through a full-scan analysis.

### 2.9. Whole Genome Sequencing of BC11-1 and Pan-Genomics Analysis

Genomic DNA was extracted from BC-11 cells using the SDS method described by Lim et al. [38]. Libraries for single-molecule real-time (SMRT) sequencing were prepared with an insert size of 10 kb using the SMRTbell™ Template kit, version 1.0 (Pacific Biosciences, Menlo Park, CA, USA), and size selection was performed using the BluePippin System. Whole genome sequencing was carried out on PacBio Sequel and Illumina NovaSeq PE150 platforms at Beijing Novogene Bioinformatics Technology Co., Ltd. Function annotation was processed by blasting genes with public databases, including the Kyoto Encyclopedia of Genes and Genomes (KEGG) and Gene Ontology (GO). Genome servers (TYGS) were used to carry out a whole genome-based taxonomic analysis [39]. Related type strains were further selected for comparative analysis. Core and specific genes were clustered using the CD-HIT rapid clustering of similar proteins software (Version 4.6.1, Open Source, Foster City, CA, USA) with a threshold of 50% pairwise identity and 0.7 length difference cutoff in amino acids. Venn diagrams were generated to illustrate relationships among the samples. Single nucleotide polymorphisms (SNPs), insertions and deletions (indels), and structural variations (SVs) were identified through genomic alignment using MUMmer (version 3.22 [40], Open Source, USA) and LASTZ (version 1.02.00, Penn State, University Park, PA, USA [41]) tools.

### 2.10. Toxicity Assessment of BC11-1

Twelve 8-week-old male C57BL/6J mice were purchased from SPF (Beijing, China) Biotechnology Co., Ltd. The mice were randomly divided into two groups (*n* = 6) after acclimatization to the local environment. Group I served as the control and received saline, while Group II was administered 5000 mg/kg of antifungal strain powder daily for 7 d. The mice were given intragastric infusions (iG) of diluted anti-aflatoxigenic strain powder in saline. After 30 d, the mice were euthanized, and the weights of their major organs were recorded. Additionally, their body weights and blood glucose levels were measured. A histopathological examination of the lung, heart, kidney, liver, and spleen was conducted using the hematoxylin and eosin staining method at Chengdu Lilai Biotechnology Co., Ltd. All animals were maintained on a standard diet with access to clean drinking water under constant conditions of 22 °C and a 12 h light/dark cycle. The testing was approved by the ethics committee at West China Hospital in China (Permit No. 20230113004), and the testing conformed to all guidelines and regulations for working with living animals.

## 3. Results

### 3.1. Anti-Aflatoxigenic Strain BC11-1 Exhibits a Broad Spectrum of Antifungal Activity but Co-Exists with Trichoderma Biocontrol Strains

**Figure 1:**
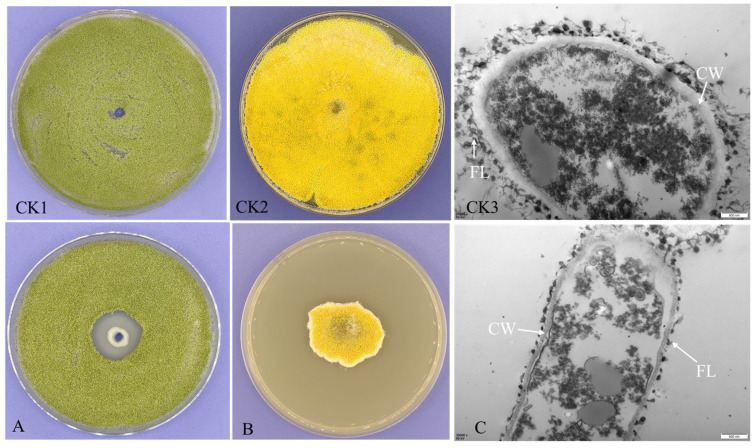
Inhibition of *A. flavus*. (**A**) Bacterial suspension of BC11-1 exhibits inhibition of the growth of *A. flavus*. (**B**) Inhibition of the growth of *A. flavus* on PDA plate containing cell-free supernatant (CFS) of BC11-1. (**C**) Alterations in the ultrastructure of *A. flavus* after treatment with the CFS of BC11-1 extract. CK1: normal growth of *A. flavus* on PDA plate; CK2: growth situation of *A. flavus* on PDA plate containing 25 mL sterile water as a negative control; CK3: normal ultrastructure of *A. flavus*; CW: cell wall; FL: fibrillar layer.

The presence of a visible halo around colonies of *A. flavus* was assessed and compared to a negative control. As a result, an anti-aflatoxigenic strain with a clear zone diameter of 2.1 cm was isolated from a woodland soil sample collected in Luzhou city (Figure 1A). The strain was designated BC11-1. When *A. flavus* was grown on PDA plates containing cell-free supernatant (CFS) from this putative anti-aflatoxigenic strain, significant inhibition of *A. flavus* growth was also observed (Figure 1B). TEM observations indicated alterations in the structure of *A. flavus* hyphae following treatment with CFS, relative to the control, including thinning of the cell wall and extensive damage to the outer fibrillar layer (Figure 1C). These results indicate that metabolites secreted by BC11-1 contribute to its antifungal properties. The morphology of BC11-1 anti-aflatoxigenic strain exhibited significant variation after 3 d of culture at 24 °C on LB and NA media (Table S1).

**Figure 2:**
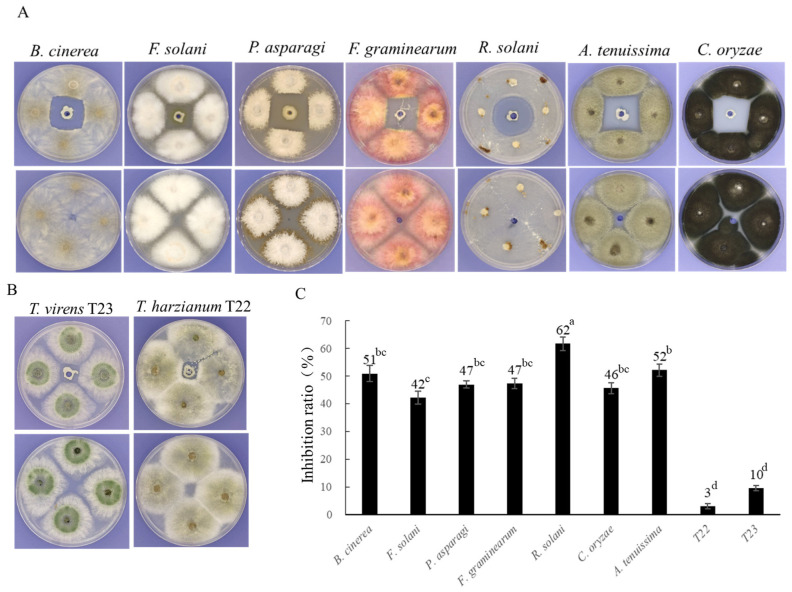
Antifungal activity of BC11-1 against several phytopathogens and biocontrol agents. (**A**) BC11-1 antifungal analysis. BC11-1 (top) demonstrated antifungal activity, relative to the negative control (bottom), against *B. cinerea*, *F. solani*, *P. asparagi*, *F. graminearum*, *R. solani*, *A. tenuissima*, and *C. oryzae*; (**B**) BC11-1 had a negligible inhibitory effect on *Trichoderma* species. (**C**) Quantitative assessment of the antifungal activity of BC11-1 against different fungi. Data represent the mean inhibition ratio (*n* = 4). Different lowercase letters above the bars indicate significant differences in the level of inhibition of the different fungi by BC11-1, relative to the control (*p* < 0.05; Tukey’s test).

BC11-1 also exhibited inhibitory activity against several other pathogenic fungi, including *Botrytis cinerea*, *Fusarium solani*, *Phomopsis asparagi*, *Fusarium graminearum*, *Rhizoctonia solani*, *Alternaria tenuissima*, and *Curvularia oryzae* (Figure 2A). Notably, BC11-1 only had a minor effect on the growth of two commonly used biocontrol fungi, *Trichoderma virens* and *Trichoderma harzianum* (Figure 2B). The inhibition ratio of the pathogenic fungi ranged from 42% to 61%, while *T. virens* T23 and *T. harzianum* T22 were only inhibited by 10% and 3%, respectively (Figure 2C).

### 3.2. Degradation of Mycotoxins by Extracellular Metabolites of BC11-1

**Figure 3:**
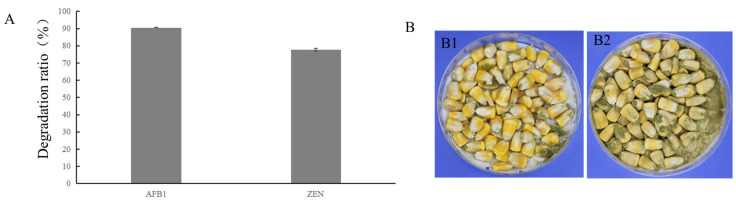
Mycotoxin detoxification activity of BC11-1. (**A**) AFB_1_ and ZEN detoxification ratio by extracellular metabolites of BC11-1. (**B**) CFS derived from BC11-1 inhibited the hyphal growth and spore formation in *A. flavus* (B1) and reduced AFB_1_ content, compared to the control (sterile water) (B2).

We determined if extracellular metabolites secreted by BC11-1 could degrade mycotoxins. BC11-1 bacterial cells were cultured on top of a 0.22 µm Millipore filter on LB medium containing AFB_1_ or ZEN mycotoxins for 7 d. The filter was used to prevent the direct contact of bacterial cells with the mycotoxin while still allowing for the exchange of metabolites. An enzyme-linked immunosorbent assay analysis revealed that the extracellular metabolites of BC11-1 had a 90% degradation ratio for AFB_1_ and a 78% degradation ratio for ZEN (Figure 3A), indicating that BC11-1 constitutively secretes mycotoxin degrative metabolites. Corn kernel assays also demonstrated that corn seeds treated with BC11-1 CFS and inoculated with *A. flavus* exhibited reduced spore formation after 7 d of incubation, relative to seeds treated with sterile water (Figure 3B). Aflatoxin levels were determined using an enzyme-linked immunosorbent assay. The concentration of AFB_1_ in corn seed samples treated with sterile water was 3936 µg/kg and 135 µg/kg in corn seeds treated with BC11-1 CFS. These data indicate strain BC11-1 significantly inhibited the growth of *A. flavus* and reduced AFB_1_ production in corn seeds.

### 3.3. Biochemical and Physiological Attributes of BC11-1

A comprehensive biochemical profile of BC11-1 was obtained using a Biolog GENIII identification MicroPlate. Table 1 lists the utilization of 71 carbon sources by BC11-1 and its sensitivity to 22 chemicals. The results indicated that BC11-1 utilized most of the examined sole carbon source sugars, except for sorbitol, mannitol, and arabinose. The optimal growth of BC11-1 was observed at pH levels up to 6.0, and BC11-1 was able to tolerate 1% NaCl. BC11-1 exhibited high sensitivity to minocycline, guanidine HCl, nalidixic acid, lithium chloride, potassium tellurite, sodium butyrate, and sodium bromate while exhibiting tolerance to 1% sodium lactate, fusidic acid, troleandomycin, rifamycin SV, lincomycin, Niaproof 4, vancomycin, tetrazolium violet, tetrazolium blue, and aztreonam.

**Figure 4:**
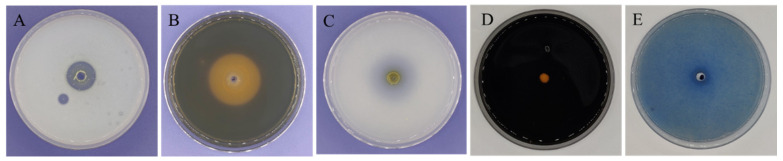
Biochemical assessment of BC11-1. A single BC11-1 colony was inoculated in 10 mL of LB broth and cultured overnight at 28 °C on a rotary shaker. Then, 50 µL of suspension was placed into holes in the center of the various assay plates. The formation of a halo surrounding the colony revealed the ability of the BC11-1 to produce extracellular protease (**A**) and a siderophore (**B**). The assays also demonstrated phosphate solubilization activity (**C**). No evidence of extracellular amylase (**D**) or β-1 and 3-glucanase (**E**) activity was observed.

Physiological attributes of BC11-1 were characterized using the hole drilling method. Results indicated that BC11-1 produces protease and a siderophore, as well as two extracellular enzymes of significant commercial value (Figure 4A,B). BC11-1 also exhibited a strong ability to solubilize phosphate, as evidenced by a clear solubilization zone around the BC11-1 colony cultured on NBRIP plates (Figure 4C). Notably, amylase and β-1 and 3-glucanase activity were not detected (Figure 4D,E).

### 3.4. Phosphate Solubilization Efficiency of BC11-1

**Figure 5:**
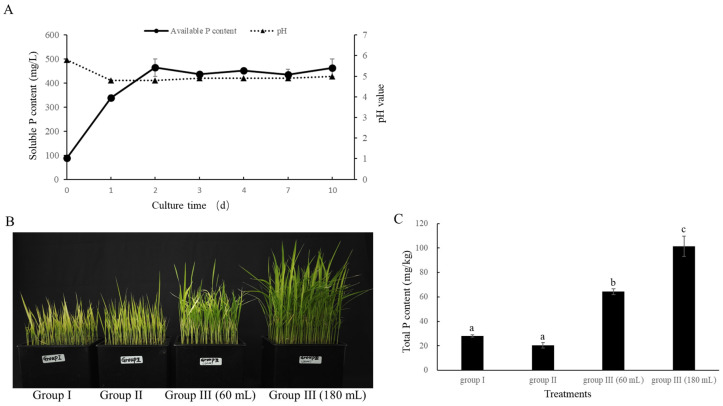
Quantitative assessment of phosphate solubilization and plant growth-promoting activity of BC11-1. (**A**) After being exposed to BC11-1 for 0, 1, 2, 3, 4, 7, and 10 d, dynamic change in soluble phosphate content in NBRP broth media and the pH value. Data represent the mean ± SD (*n* = 3). Data represent the mean ± SD (*n* = 3). (**B**) Three groups of rice seedlings were subjected to different treatments (from left to right), as follows: Group I received tap water only; Group II received tap water containing 5 g/L Ca_3_(PO_4_)_2_; and Group III received tap water containing either 60 mL or 180 mL phosphate solution produced by BC11-1. (**C**) Phosphate content in plants in the three different groups. Data represent the mean ± se (*n* = 3). Different lowercase letters above the bars indicate significant differences in the level of phosphorus in plants in the three different groups of fungi (*p* < 0.05) as indicated by a Tukey’s test.

A distinct solubilization halo was observed around the BC11-1 colony on the NBRIP plate, indicating the potential use of BC11-1 as a phosphate-solubilizing bacterium. Therefore, the level of soluble phosphate in the NBRIP liquid medium treated with BC11-1 was assessed after 0, 1, 2, 3, 4, 7, and 10 d to further confirm the phosphate-solubilizing capacity of BC11-1. The results indicated that the content of soluble phosphate markedly rose to 338 mg/L on the first day of culture and peaked at 464 mg/L the following day, with no significant change observed over the subsequent 8 d. These data confirm that BC11-1 effectively converts insoluble tricalcium phosphate into soluble phosphate (Figure 5A). The pH of the medium also decreased from an initial value of 5.8 to 4.8 after treatment with BC11-1 on day 1 and remained stable between pH 4.8 and 4.9 from day 2 to day 10 (Figure 5A).

A greenhouse experiment was conducted on rice seedlings grown hydroponically, utilizing three distinct groups of seedlings to assess the uptake and utilization of phosphate solubilized by BC11-1. The results revealed that seedlings in Groups I (treated with tap water only) and II (treated with tap water containing 5 g/L Ca_3_(PO_4_)_2_) exhibited signs of poor health, stunted growth, and yellowing after 14 d of growth. These symptoms are indicative of significant nutrient deficiencies. In contrast, seedlings in Group III (treated with tap water containing either 60 mL or 180 mL of phosphate solution produced by BC11-1) appeared notably healthier and greener compared to seedlings in Groups I and II. A positive correlation was also observed between the amount of phosphate solution added and the growth of rice seedlings (Figure 5B). The total P content in the rice plants, including the roots, was measured using the ICP-OES method to confirm the uptake and utilization of phosphorus (P) in rice seedlings. The results indicated that the total P content in seedlings from Group III was significantly higher than it was in seedlings from Groups I and II. Additionally, the use of a higher amount of BC11-1-digested Ca_3_(PO_4_)_2_ solution was reflected in an increased level of P content in Group III rice plants (Figure 5C). These findings confirm the ability of BC11-1 to convert calcium phosphate into soluble phosphate, which can be absorbed and utilized by seedlings to enhance growth.

### 3.5. BC11-1 Produces Cytokinin

**Figure 6:**
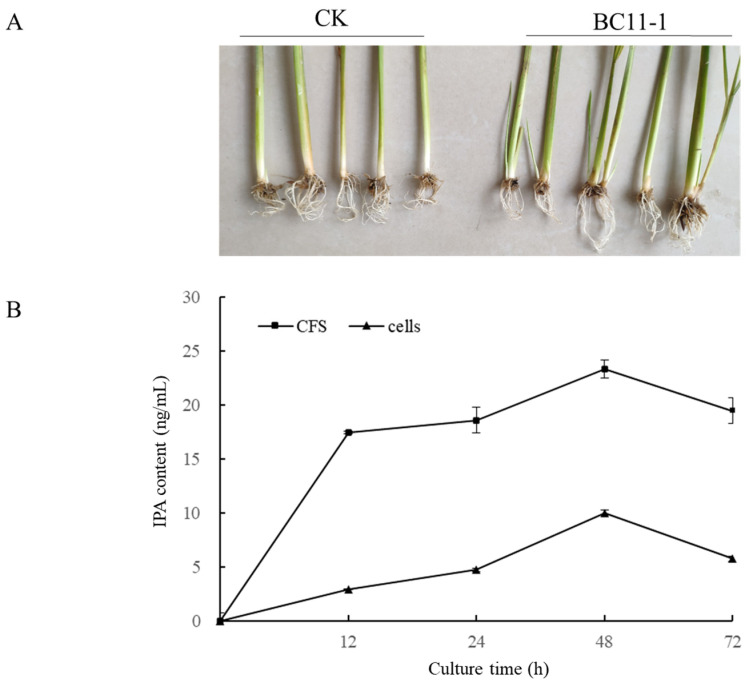
Analysis of cytokinin production by BC11-1 in cells and CFS. (**A**) Promotion of tillering promotion by CFS of BC11-1. CK: rice seedlings treated with water as negative control. (**B**) Isopentenyl adenosine (IPA) in BC11-1 cells and cell-free supernatant (CFS) over culture time. Data represent the mean ± se (*n* = 3).

Early tillering was observed in rice seedlings treated with BC11-1 solution during the greenhouse experiment (Figure 6A); we suspected that BC11-1 may produce cytokinin, which would promote tillering. Therefore, we utilized an HPLC-MS analysis to determine the presence of cytokinin in BC11-1 CFS and cells. The analysis demonstrated the presence of isopentenyl adenosine (IPA) in both BC11-1 CFS and cells. The levels of IPA in BC11-1 CFS and cells gradually increased during the early stages of culture, reaching peak concentrations of 23 ng/mL and 10 ng/mL at 48 h, respectively, followed by a significant decline in both CFS and cells after 72 h of culture (Figure 6B).

### 3.6. Genome Analysis

An intensive study of whole genome analysis of BC11-1 is of important value for functional gene mining. According to the whole genome sequencing results, the genome of BC11-1 was assembled to be approximately 8,317,431 bp, with GC content of 66% (accession number: NMDC60146102, https://nmdc.cn/resource/genomics/genome/detail/NMDC60146102, access on 16 January 2024), and a total of 7657 genes were identified. Specifically, 4979 genes were annotated in the GO database, and 7227 genes were annotated in the KEGG database. Notably, in the KEGG database, 149 functional genes were associated with xenobiotics biodegradation and metabolism (Figure S1). Based on the genome server (TYGS) result, 14 strains were found with a closely related genome sequence for the anti-aflatoxigenic bacteria BC11-1; the nearest match strain was the *Burkholderia contaminans*.

**Figure 7:**
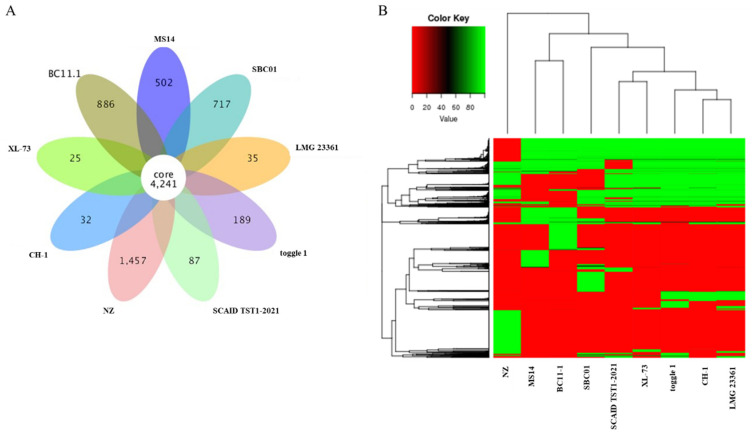
Pan-genome analysis. (**A**) Venn diagram indicating the core gene number (common to all strains) and strain-specific gene number in eight *B. contaminans* strains, including four biocontrol strains, MS14 (GenBank: ASM102914v1), CH1 (GenBank: ASM472362v1), NZ (GenBank: ASM336316v1), and XL-73 (GenBank: ASM975562v1), and four pathogenic strains, toggle 1 (GenBank: ASM1822378v1), SCAID TST1-2021 (GenBank: ASM1991536v1), LMG 23361 (GenBank: ASM175838v2), and SBC01 (GenBank: ASM1688794v1). (**B**) Dispensable gene heat map (left, dispensable gene cluster; top, strain cluster; Colors represent the identity of dispensable genes in the eight *B. contaminans* strains).

A comparative genomics analysis was conducted using eight previously sequenced *Burkholderia contaminans* strains (Table S2), including four biocontrol strains, MS14 (GenBank: ASM102914v1), CH1 (GenBank: ASM472362v1), NZ (GenBank: ASM336316v1), and XL-73 (GenBank: ASM975562v1), and four pathogenic strains, toggle 1 (GenBank: ASM1822378v1), SCAID TST1-2021 (GenBank: ASM1991536v1), LMG 23361 (GenBank: ASM175838v2), and SBC01 (GenBank: ASM1688794v1). The comparative genomic analysis included the identification of core genes, specific genes, SNPs, indels, and SVs. The results revealed that the pan-genome of these eight *B. contaminans* strains comprised 12,416 genes, with approximately 4241 genes predicted to constitute the core genome and 3930 genes being strain-dependent. Notably, the genomic analysis identified 886 specific genes unique to BC11-1 (Figure 7A). A dispensable gene analysis indicated that the dispensable genes in BC11-1 clustered together with those in the MS14 biocontrol strain of *B. contaminans* (Figure 7B). A comparison of BC11-1 and the MS14 biocontrol strain genomes revealed only 49,713 SNPs (Table 2), with only five coding DNA sequences (CDS) in BC11-1 and another biocontrol strain, NZ exhibiting indels (Table 3). Collectively, the structural aspects and gene content of BC11-1 support its classification as a potential biocontrol strain rather than a pathogenic strain.

### 3.7. Assessment of Animal Safety

A mouse model was employed to assess the impact of BC11-1 on animal health. BC11-1 powder mixed with saline solution was administered to mice via intragastric infusion (iG) and directly delivered to the stomach for seven consecutive days. The body weight and blood glucose levels of the iG mice were monitored at 7 d intervals over a period of four weeks. After 30 d, no significant differences were observed in body weight and blood glucose levels between the iG mice and those treated with saline alone (Table S3). Three days after intragastric infusion, heart, liver, spleen, lung, and kidney tissues were harvested from some iG mice and saline-treated mice for histological examination. An analysis of the tissues revealed no discernible pathological alterations in the iG mice compared to the saline-treated mice (Figure S2). These findings provide evidence that BC11-1 does not pose a risk to animal health.

## 4. Discussion

Aflatoxin B_1_ (AFB_1_) contamination poses a significant threat to human and animal health in China and has been primarily attributed to *A. flavus*, the predominant AFB_1_-producing fungus. Biological control methods provide an environmentally friendly approach to mitigate aflatoxin contamination by targeting the growth of aflatoxigenic *A. flavus* [42]. Therefore, our initial focus was on identifying potential anti-aflatoxigenic strains to develop biocontrol agents for controlling *A. flavus* and aflatoxin contamination. Hundreds of isolates were evaluated in our previous research for their ability to counteract aflatoxigenic *A. flavus*, with only a few demonstrating substantial and persistent antagonistic effects. Among the evaluated strains, *B. contaminans* BC11-1 exhibited a high level of efficacy. *Burkholderia* species are known for their adaptability to diverse environmental conditions and are commonly found in the rhizosphere of plants, in which they play a variety of roles, including biocontrol of plant pathogens and promotion of plant growth, particularly in nutrient-poor environments [43,44,45]. *B. contaminans* is prevalent in natural habitats and possesses several distinctive characteristics. Previous studies have demonstrated the broad antifungal activity of *B. contaminans* strains, such as MS14, against various plant soilborne fungal pathogens [46]. *B. contaminans* strain B-1 has also been shown to effectively control postharvest diseases in strawberry fruits [47]. In the present study, we demonstrate that *B. contaminans* BC11-1 is an anti-aflatoxigenic strain that exhibits broad-spectrum antifungal activity, as well as the ability to degrade AFB_1_ and ZEN mycotoxins, with degradation ratios of 90% and 78%, respectively. Our findings indicate that extracellular metabolites play a crucial role in the anti-aflatoxigenic and mycotoxin detoxification activity of BC11-1. Occidiofungin has been reported to be a unique antifungal glycopeptide produced by *B. contaminans* MS14 [48]. We hypothesize that *B. contaminans* strain BC11-1 may synthesize homologs of natural antifungal products, although the specific metabolites responsible for mycotoxin detoxification are still unknown. An objective of our continuing research is to comprehensively assess the detoxification spectrum of BC11-1 and identify the compounds involved in detoxification. While some bacterial species, including lactic acid bacteria and strains of *Lysinibacillus* sp., have demonstrated mycotoxin detoxification capacity, only a limited number have exhibited antifungal activity against mycotoxin-producing fungi, or vice versa [9,49,50]. Therefore, we suggest that BC11-1 represents a promising resource for preventing mycotoxin preharvest and postharvest contamination.

As a PGPR, strain BC11-1 also exhibited multiple functions, including phosphorus solubilization ability and cytokinin production. In this study, we found that BC11-1 was able to solubilize more than 450 mg/L soluble phosphate from tricalcium phosphate after it was cultured for 48 h in NBRIP broth, and the soluble phosphate converted from calcium phosphate can be absorbed and utilized by rice seedlings to enhance growth. The amount of phosphate solubilized by BC11-1 was higher compared to other previously reported phosphate-solubilizing strains. Wan et al. [51] isolated 18 phosphate-solubilizing strains from eight different genera in soil using a stepwise acclimation strategy, each exhibiting a different capacity for solubilizing different forms of insoluble phosphorus. However, even after 5 d of incubation in NBRIP broth, their soluble phosphorus content remained below 300 mg/L. Although some phosphate-solubilizing strains have demonstrated robust phosphorus solubilization ability when the soluble-to-insoluble phosphorus ratio exceeded 700 mg/L, they did not show any antifungal or mycotoxin detoxification ability [52,53]. Previously, a biocontrol *B. contaminans* strain identified as KNU17BI1 was found to solubilize 300 mg/L of phosphorus from tricalcium phosphate after 3 d of incubation, reaching a peak of 491 mg/L on the 10th day [54]. As it turns out, the phosphorus solubilization efficiency of BC11-1 was equal to the reported efficiency of *B. contaminans* KNU17BI1. Hence, the development of commercial biofertilizers would greatly benefit from the considerable phosphorus solubilization ability of BC11-1.

Phytohormones synthesized and secreted by bacteria, including cytokinin, gibberellin, and auxin, modify the phytohormone pool present in the soil environment and can promote plant growth and development [55,56]. The potential of *Burkholderia* species in phytohormone production has been previously assessed. Studies have demonstrated that most of the tested *Burkholderia* sp. strains produce IAA and modulate the L-tryptophan-dependent synthesis pathway [57,58]. KNU17BI1 represents the first reported strain of *B. contaminans* recognized for its notable production of IAA, yielding a substantial amount of IAA (60 µg/mL) when the L-tryptophan concentration was 2 mg/mL. However, it failed to produce IAA in the absence of L-tryptophan [54]. Notably, we observed no evidence of IAA production in either the CFS or bacterial cells of *B. contaminans* BC11-1, regardless of the presence of L-tryptophan. Instead, our analysis confirmed that *B. contaminans* BC11-1 produces cytokinin, as evidenced by the detection of IPA in the CFS and cells. These findings highlight the significant variations that exist in the properties of different strains of bacteria within the same species. Cytokinin is described to be involved in stimulating plant cell division and increasing abiotic stress tolerance. Several bacteria can synthesize cytokinin. Cytokinin production by bacteria can enhance plant vigor and resilience under adverse conditions [56,59], making cytokinin a highly desirable trait for bioagents intended for use in challenging soil environments. Overall, *B. contaminans* BC11-1 exhibits several beneficial, multifunctional properties, further confirming its value as a biocontrol agent capable of enhancing soil fertility, promoting growth, and providing plant protection.

Comparative genomic analysis provides insights into the ecological diversity and evolutionary history of microorganisms. The genomic variability observed among multiple strains of the same species can potentially reveal that the biological properties of a species have been underestimated and that the true potential is represented in the pan-genome. This concept indicates that the sequence of a single genome does not fully capture the genetic variability driving microorganisms within a species [60]. Studies suggest that the bacterial pan-genome may influence gene essentiality, with essential genes potentially evolving to become non-essential, although their level of dispensability may vary based on genetic background and/or environmental factors [61]. It is reported that *Burkholderia cepacia* complex (Bcc) is a group of closely related, remarkably versatile bacteria found in natural environments, and the Bcc group is composed of at least 18 different species, including *B. contaminans* [62]. Therefore, in the present study, we analyzed and compared the genome sequence of multiple *B. contaminans* strains. The results indicated that the pan-genome of *B. contaminans* comprises 12,416 genes, with only 4241 represented in a common, core genome. *B. contaminans* BC11-1 was found to cluster with other recognized biocontrol strains. The biosafety of BC11-1 was also assessed in animal tests. Future studies will explore if strain-specific genes are essential or non-essential. Notably, the identification of 886 BC11-1-specific genes suggests that other biological properties of BC11-1 remain to be discovered.

## 5. Conclusions

In the present study, we have isolated an anti-aflatoxigenic *B. contaminans* strain, BC11-1, conferring a broad spectrum of antifungal activity and mycotoxin detoxification ability, either with living cells or CFS. Moreover, we have revealed its high ability to solubilize phosphorus and produce cytokinin production. The multifunctional characteristics of BC11-1 highlight its potential as a biocontrol agent and/or a biofertilizer. Finally, 886 BC11-1-specific genes have been revealed through a pan-genomic analysis, suggesting that BC11-1 may possess other undiscovered biological properties. *B. contaminans* belongs to a group of closely related bacteria called the “*Burkholderia cepacia* complex (Bcc)”. In view of the controversy of Bcc bacteria, we will try to mine functional genes or gene clusters of BC11-1 based on its genomic information in the next study and understand its synthesis regulation mechanisms of functional metabolites.

## Figures and Tables

**Table 1:** Biochemical characteristics of BC11-1.

Carbon Sources Utilization	Result	Carbon Sources Utilization	Result	Carbon Sources Utilization	Result	Biochemical Characteristics Test	Result
D-Glucose	++	Stachyose	−	Mucic Acid	+	pH6	+++
D-Mannose	++	Gelatin	−	Quinic Acid	++	pH5	++
D-Fructose	++	Pectin	−	D-Saccharic Acid	++	1% NaCl	++
D-Galactose	++	Tween 40	−	p-Hydroxy Phenylacetic Acid	−	4% NaCl	−
3-Methyl Glucose	−	β-Methyl-D-Glucoside	−	Methyl Pyruvate	−	8% NaCl	−
D-Fucose	++	D-Salicin	−	D-Lactic Acid Methyl Ester	−	1% Sodium Lactate	+++
L-Fucose	++	N-Acetyl-D-Glucosamine	−	L-Lactic Acid	+	Fusidic Acid	+++
L-Rhamnose	+	D-Glucose-6-PO_4_	+++	Citric Acid	+	Troleandomycin	+++
D-Sorbitol	−	D-Fructose-6-PO_4_	+++	α-Keto-Glutaric Acid	−	Rifamycin SV	+++
D-Mannitol	−	D-Aspartic Acid	−	D-Malic Acid	−	Minocycline	−
D-Arabitol	−	D-Serine	++	L-Malic Acid	++	Lincomycin	+++
myo-Inositol	+	Glycyl-L-Proline	−	Bromo-Succinic Acid	−	Guanidine HCl	−
Glycerol	−	L-Alanine	+	γ-Amino-Butryric Acid	++	Niaproof 4	+++
D-Maltose	−	L-Arginine	+	α-Hydroxy Butyric Acid	−	Vancomycin	+++
D-Trehalose	−	L-Aspartic Acid	−	β-Hydroxy-D,L Butyric Acid	−	Tetrazolium Violet	+++
D-Cellobiose	−	L-Glutamic Acid	+	α-Keto-Butyric Acid	−	Tetrazolium Blue	+++
Gentiobiose	−	L-Histidine	++	Acetoacetic Acid	−	Nalidixic Acid	−
Sucrose	−	L-Pyroglutamic Acid	+	Propionic Acid	−	Lithium Chloride	−
D-Turanose	−	L-Serine	−	Acetic Acid	++	Potassium Tellurite	−
α-D-Lactose	−	D-Galacturonic Acid	+++	Formic Acid	−	Aztreonam	++
D-Melibiose	−	L-Galactonic Acid Lactone	+++	N-Acetyl-β-D Mannosamine	−	Sodium Butyrate	−
D-Raffinose	−	D-Gluconic Acid	++	N-Acetyl-D-Galactosamine	−	Sodium Bromate	−
Dextrin	−	D-Glucuronic Acid	+++	N-Acetyl Neuraminic Acid	−		
Inosine	−	Glucuronamide	+++				

Notes: “+”, Positive; “+++”, Strongly positive; “++”, Moderate positive; “−”, Negative.

**Table 2:** Comparative SNP analysis of BC11-1 with other *B. contaminans* strains.

Reference Strain	Sample Strain	Start Syn	Stop Syn	Start Nonsyn	Stop Nonsyn	Premature Stop	Synonymous	Nonsynonymous	Total CDS SNP	Intergenic	Total SNP
MS14	BC11-1	2	36	14	10	32	29,645	10,559	40,285	9428	49,713
NZ	BC11-1	29	130	96	91	267	82,245	38,908	121,623	32,390	154,013
CH1	BC11-1	4	115	51	42	91	88,455	30,610	119,319	31,884	151,203
XL-73	BC11-1	5	113	52	43	93	88,209	30,527	118,998	31,908	150,906
SBC01	BC11-1	8	128	53	44	108	89,828	30,492	120,579	32,476	153,055
LMG 23361	BC11-1	2	108	51	38	80	81,480	28,223	109,934	28,754	138,688
toggle 1	BC11-1	5	113	52	47	104	88,096	30,969	119,337	32,143	151,480
SCAID TST1-2021	BC11-1	6	105	52	45	126	82,822	30,371	113,440	30,181	143,621

Notes: genome sequences of other 7 strains were used as references when compared with that of BC11-1; syn stands for synonymous; nonsyn stands for nonsynonymous.

**Table 3:** Comparative indel analysis between BC11-1 and other *B. contaminans* strains.

Reference Strain	Sample Strain	All CDS	CDS with Indel	Frame-Shifted	Start Codon	Stop Codon	Premature Stop
MS14	BC11-1	7655	23	8	0	2	0
NZ	BC11-1	9522	5	3	0	1	0
CH1	BC11-1	7981	24	8	0	0	0
XL-73	BC11-1	7763	23	7	0	0	0
SBC01	BC11-1	8055	25	10	0	0	1
LMG 23361	BC11-1	9373	36	15	0	0	1
toggle 1	BC11-1	8446	45	11	0	0	0
SCAID TST1-2021	BC11-1	7532	79	25	1	4	4

Notes: genome sequences of other 7 strains were used as references when compared with that of BC11-1.

## Data Availability

The ethics committee at West China Hospital in China (permit no. 20230113004) approved this study’s protocol. The ethics committee approved this study on 13 January 2023. All the animal experiments in this study followed the regulations for administering affairs concerning experimental animals of Sichuan Province, China.

## Supplementary Materials

The following supporting information can be downloaded at: https://www.mdpi.com/article/10.3390/microorganisms12091754/s1, Figure S1: KEGG pathway annotation of BC11-1; Figure S2: Histopathological observations of mice treated with powdered preparation of BC11-1 cells or saline solution; Table S1: morphology characteristic of BC11-1 on different mediums; Table S2: the information of eight *Burkholderia contaminans* strains; Table S3: Organ indices in mice in control and iG groups (x ± S, *n* = 6).
