# Supplementary material for: Anti-Aflatoxigenic Burkholderia contaminans BC11-1 Exhibits Mycotoxin Detoxification, Phosphate Solubilization, and Cytokinin Production
Source: Microorganisms. 2024 Aug 23;12(9):1754. doi: 10.3390/microorganisms12091754 (PMC11434526; doi:10.3390/microorganisms12091754)
Supplement: Supplementary file 1 [file microorganisms-12-01754-s001.zip › microorganisms-3154845-supplementary/supplementary files/Fig.S2 Histopathological examination of mice..pptx]

## Slide 1
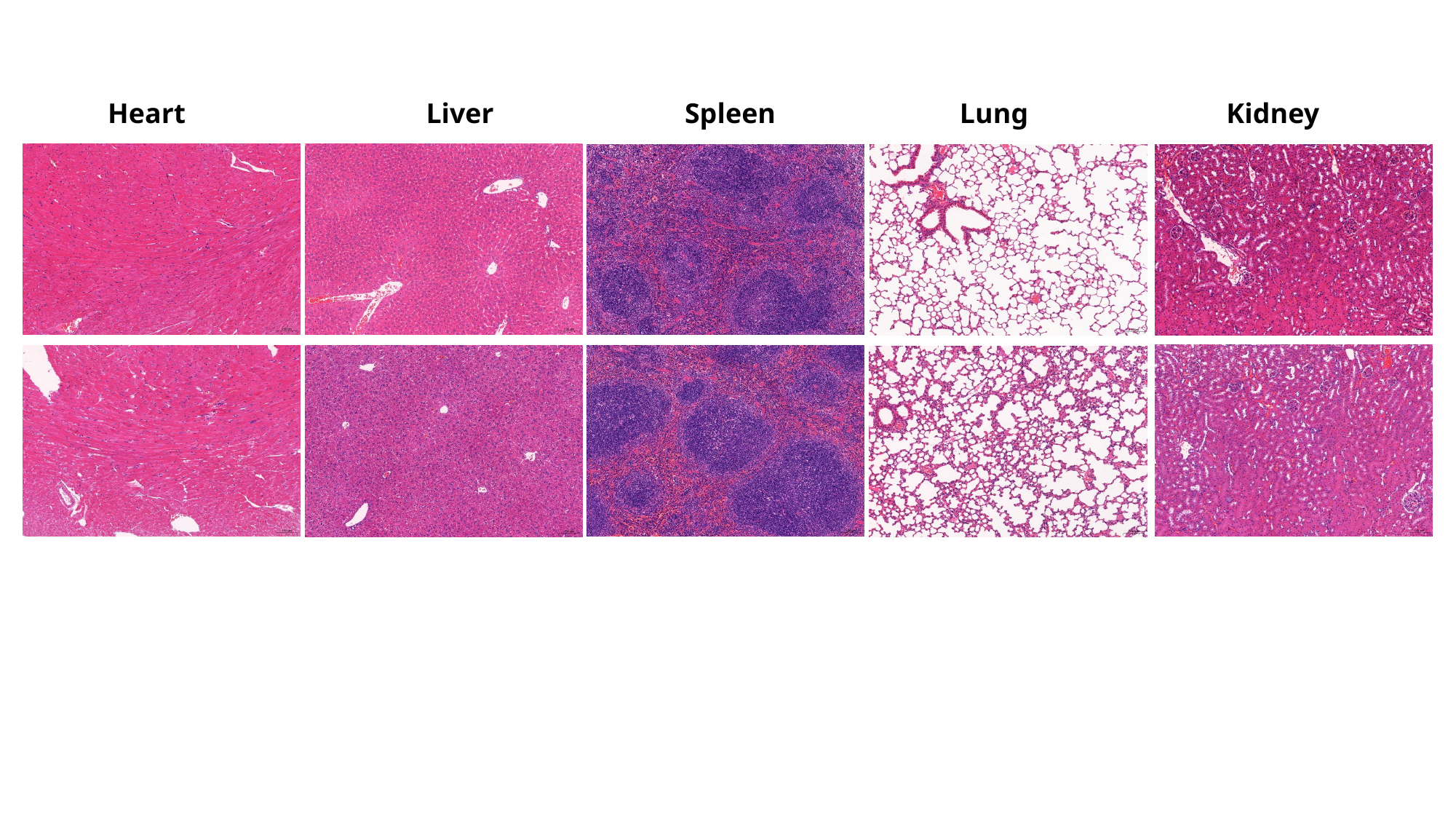

Heart Liver Spleen Lung Kidney

## Slide 2
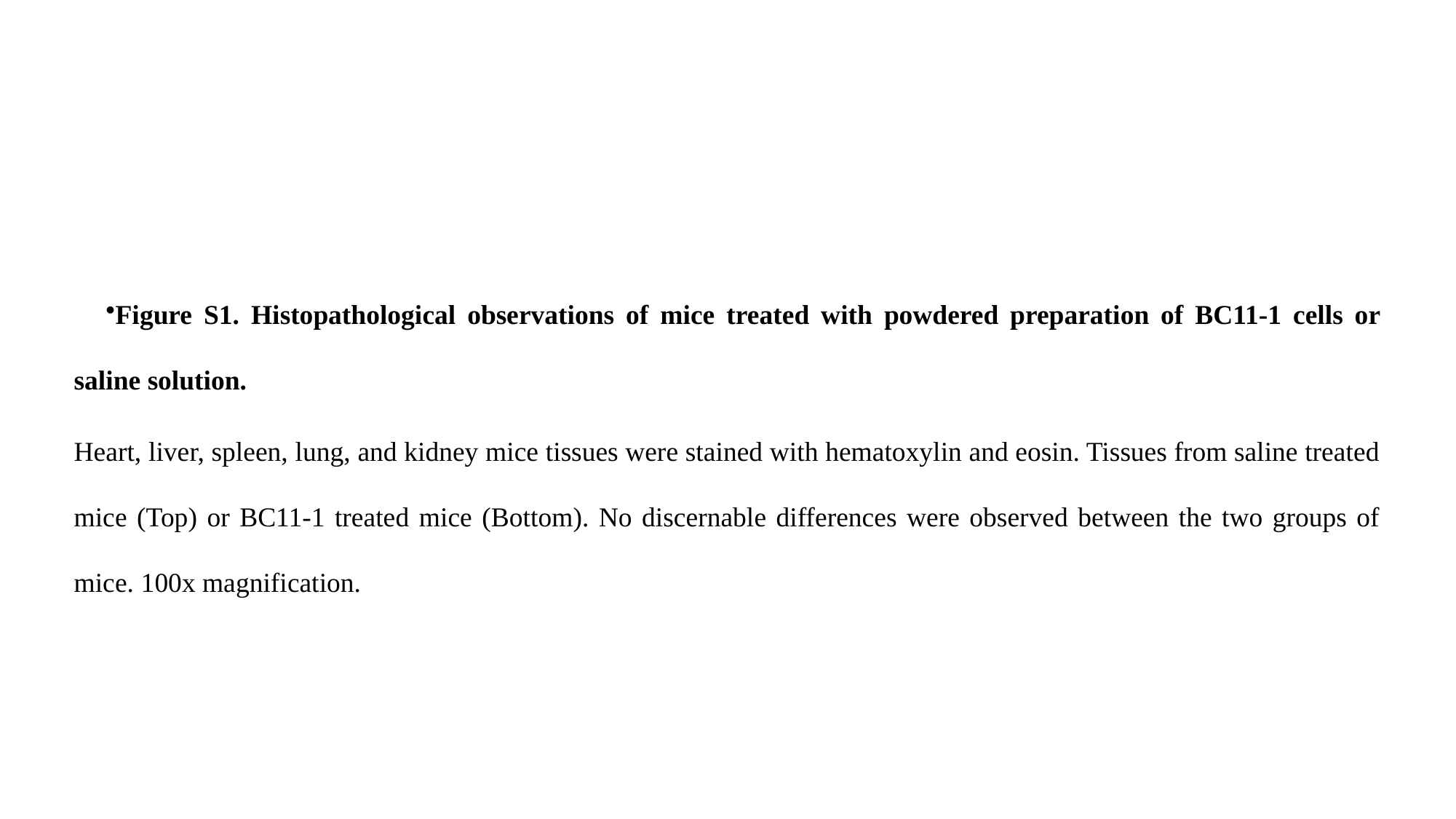

Figure S1. Histopathological observations of mice treated with powdered preparation of BC11-1 cells or saline solution.
Heart, liver, spleen, lung, and kidney mice tissues were stained with hematoxylin and eosin. Tissues from saline treated mice (Top) or BC11-1 treated mice (Bottom). No discernable differences were observed between the two groups of mice. 100x magnification.
