# Supplementary material for: Anti-Aflatoxigenic Burkholderia contaminans BC11-1 Exhibits Mycotoxin Detoxification, Phosphate Solubilization, and Cytokinin Production
Source: Microorganisms. 2024 Aug 23;12(9):1754. doi: 10.3390/microorganisms12091754 (PMC11434526; doi:10.3390/microorganisms12091754)
Supplement: Supplementary file 1 [file microorganisms-12-01754-s001.zip › microorganisms-3154845-supplementary/supplementary files/Table S1 morphology characteristic of BC11-1.pdf]

Table S1 morphology characteristic of BC11-1 on different mediums

Medium	Growth	Colony shape	Colony color	Phenotype
LB plate	slow	circular form, raised elevation, entire margin, dull surface, opaque	yellow	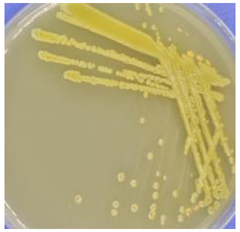 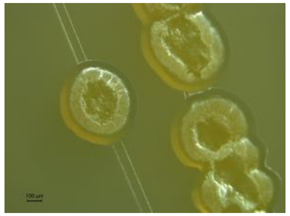
NA plate	fast	circular form, dull surface, entire margin, dull surface, opaque	creamy white	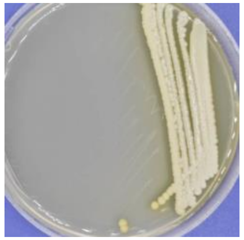 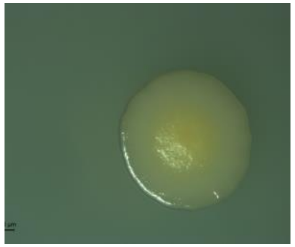
