# Supplementary material for: Anti-Aflatoxigenic Burkholderia contaminans BC11-1 Exhibits Mycotoxin Detoxification, Phosphate Solubilization, and Cytokinin Production
Source: Microorganisms. 2024 Aug 23;12(9):1754. doi: 10.3390/microorganisms12091754 (PMC11434526; doi:10.3390/microorganisms12091754)
Supplement: Supplementary file 1 [file microorganisms-12-01754-s001.zip › microorganisms-3154845-supplementary/supplementary files/Table S2 The information of eight Burkholderia contaminans strains.pdf]

Table S2 The information of eight *Burkholderia contaminans* strains

Strain name	GenBank accession number	Sample type	Collection date
MS14	ASM102914v1	soil	Jun 17, 2015
CH1	ASM472362v1	airborne-contaminated nutrient medium	Sep 8, 2017
NZ	ASM336316v1	seed of *Corchorus olitorius*	Aug 4, 2018
XL-73	ASM975562v1	cucumber rhizosphere soil	Dec 16, 2019
toggle 1	ASM1822378v1	blood	May 2, 2021
SCAID TST1-2021	ASM1991536v1	Swab from tracheostomic tube	Sep 9, 2021
LMG 23361	ASM175838v2	milk of mastitis dairy sheep	Jan 24, 2017
SBC01	ASM1688794v1	corneal scrapings	May, 2015
