# Supplementary material for: Anti-Aflatoxigenic Burkholderia contaminans BC11-1 Exhibits Mycotoxin Detoxification, Phosphate Solubilization, and Cytokinin Production
Source: Microorganisms. 2024 Aug 23;12(9):1754. doi: 10.3390/microorganisms12091754 (PMC11434526; doi:10.3390/microorganisms12091754)
Supplement: Supplementary file 1 [file microorganisms-12-01754-s001.zip › microorganisms-3154845-supplementary/supplementary files/Table S3 Organ index of mice in control and iG groups.pdf]

Table S3 Organ indices in mice in control and iG groups（$\bar{x}$±S， n = 6）

Groups	Body weight (g)	Blood glucose (mg/dl)	Organ index (%)				
			Heart	Liver	Spleen	Lung	Kidney
CK	24.22±1.64[a]	12.10±1.72[a]	0.71±0.106[a]	5.90±0.736[a]	0.30±0.0789[a]	0.62±0.0291[a]	1.42±0.0959[a]
IG	24.49±0.94[a]	13.23±1.44[a]	0.69±0.101[a]	5.62±0.514[a]	0.33±0.0796[a]	0.62±0.0479[a]	1.37±0.0638[a]

Values presented are means ± standard deviation (*n* = 6/group). Mean value followed by the same letters in each column do note differ significantly (Tukey’s test)
