# Supplementary figures and images for: Anti-Aflatoxigenic Burkholderia contaminans BC11-1 Exhibits Mycotoxin Detoxification, Phosphate Solubilization, and Cytokinin Production
Source: Microorganisms. 2024 Aug 23;12(9):1754. doi: 10.3390/microorganisms12091754 (PMC11434526; doi:10.3390/microorganisms12091754)

## Slide 1
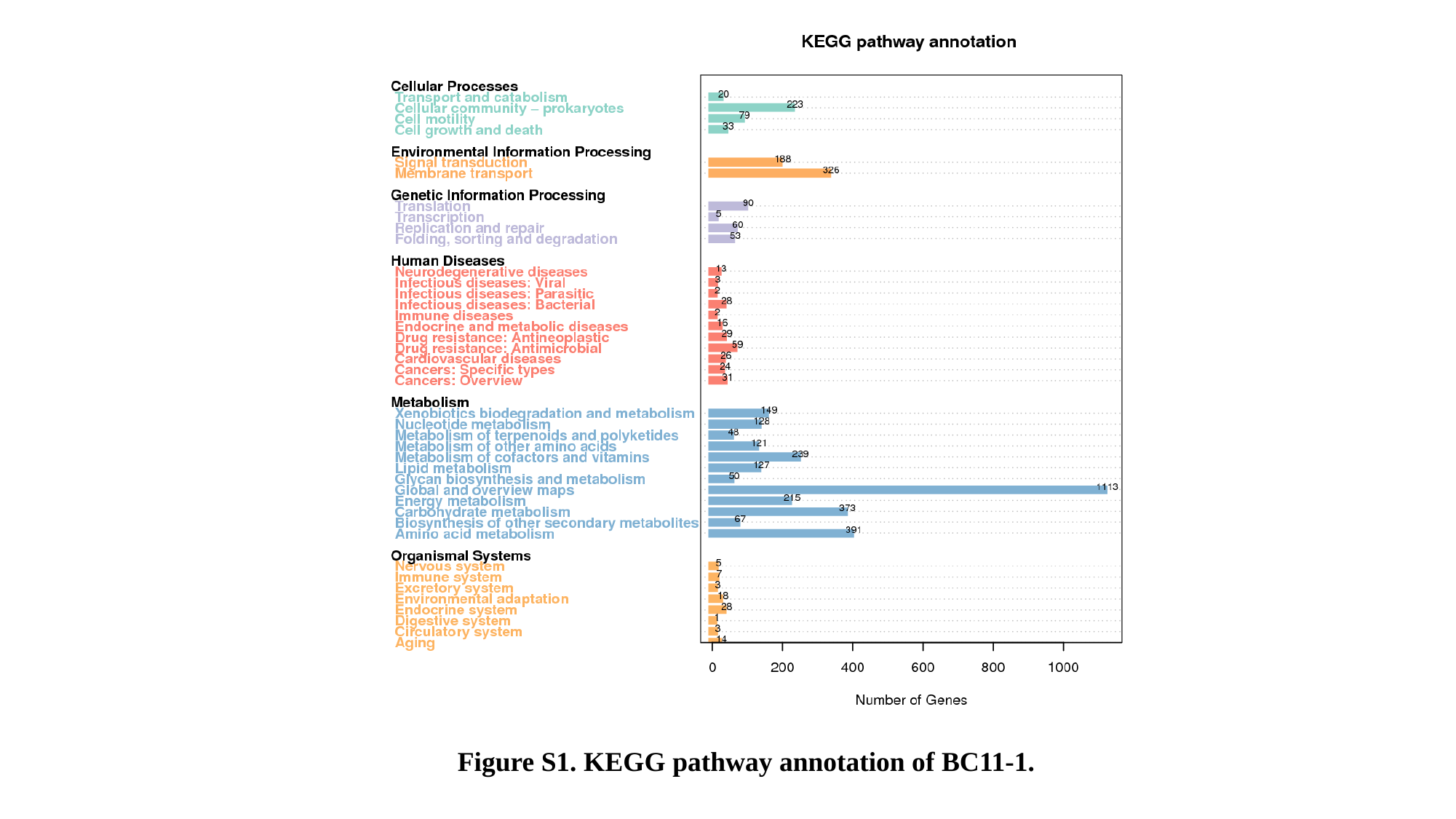

Figure S1. KEGG pathway annotation of BC11-1.

Supplement: Supplementary file 1 [file microorganisms-12-01754-s001.zip › microorganisms-3154845-supplementary/supplementary files/Fig.S1 KEGG pathway annotation of BC11-1.pptx]
